# Test–retest reliability of new and conventional echocardiographic parameters of left ventricular systolic function

**DOI:** 10.1007/s00392-018-1363-7

**Published:** 2018-10-27

**Authors:** Tomasz Baron, Lars Berglund, Eva-Maria Hedin, Frank A. Flachskampf

**Affiliations:** 1 Department of Medical Sciences, Cardiology and Clinical Physiology, Uppsala University, Uppsala, Sweden; 2 Department of Radiology, Uppsala University Hospital, Uppsala, Sweden; 3 Uppsala Clinical Research Center, Uppsala University, Uppsala, Sweden; 4 Department of Public Health and Caring Sciences/Geriatrics, Uppsala University, Uppsala, Sweden

**Keywords:** Left ventricular function, Global longitudinal strain, Ejection fraction, Mitral annulus plane systolic excursion, Test–retest reliability

## Abstract

**Background:**

Reliability of left ventricular function measurements depends on actual biological conditions, repeated registrations and their analyses.

**Objective:**

To investigate test–retest reliability of speckle-tracking-derived strain measurements and its determinants compared to the conventional parameters, such as ejection fraction (EF), LV volumes and mitral annular plane systolic excursion (MAPSE).

**Methods:**

In 30 patients with a wide range of left ventricular function (mean EF 46.4 ± 16.4%, range 14–73%), standard echo views were acquired independently in a blinded fashion by two different echocardiographers in immediate sequence and analyzed off-line by two independent readers, creating 4 data sets per patient. Test–retest reliability of studied parameters was calculated using the smallest detectable change (SDC) and a total, inter-acquisition and inter-reader intra-class correlation coefficient (ICC).

**Results:**

The smallest detectable change normalized to the mean absolute value of the measured parameter (SDCrel) was lowest for MAPSE (10.7%). SDCrel for EF was similar to GLS (14.2 and 14.7%, respectively), while SDCrel for CS was much higher (35.6%). The intra-class correlation coefficient was excellent (> 0.9) for all measures of the left ventricular function. Intra-patient inter-acquisition reliability (ICCacq) was significantly better than inter-reader reliability (ICCread) (0.984 vs. 0.950, *p* = 0.03) only for EF, while no significant difference was observed for any other LV function parameter. Mean intra-subject standard deviations were significantly correlated to the mean values for CS and LV volumes, but not for the other studied parameters.

**Conclusions:**

In a test–retest setting, both with normal and impaired left ventricular function, the smallest relative detectable change of EF, GLS and MAPSE was similar (11–15%), but was much higher for CS (35%). Surprisingly, reliability of GLS was not superior to that of EF. Acquisition and reader to a similar extent influenced the reliability of measurements of all left ventricular function measures except for ejection fraction, where the reliability was more dependent on the reader than on the acquisition.

## Introduction

Evaluation of left ventricular (LV) function remains a crucial issue in clinical decision-making and risk stratification across different cardiac disorders. Recently introduced speckle-tracking-derived measures of myocardial deformation, mainly the global peak systolic longitudinal strain (GLS), have emerged as a new standard in the assessment of LV function, with the potential of complementing or even replacing ejection fraction (EF) due to the well-recognized examiner-dependency of the latter [1–4]. GLS has been reported to be superior to EF in the detection of incipient heart failure of different etiologies, for example the cardio toxic effects of cancer therapy [5, 6], aortic stenosis [7] or amyloidosis [8]. Since GLS is a semi-automatically generated parameter, it might be less affected by user input. However, although the image processing is largely performed by machine and software, image acquisition continues to be operator-dependent.

Evaluation of the test–retest reliability in a real-life clinical setting is crucial to distinguish the effects of the disease (biological variability) from the variability of the measurement. The variability of the measurement depends both on acquisition and reading variability and therefore is more complex, but also more realistic than inter-observer or intra-observer variability, which focus on repeated analyses of the same not-repeated registrations. Much more limited data exist on the test–retest reliability of functional left ventricular parameters than on their observer variability.

The purpose of this study was to investigate test–retest reliability of speckle-tracking-derived LV strain measurements (global peak systolic longitudinal strain (GLS) and circumferential strain (CS)) and its determinants in relation to the conventional parameters, such as ejection fraction (EF) calculated from end-diastolic (EDV) and end-systolic volumes (ESV), and mitral annular plane systolic excursion (MAPSE), in a prospective study of patients with a wide range of left ventricular function.

## Materials and methods

A total of 30 patients in sinus rhythm with different degrees of LV function impairment were prospectively recruited in the study. All the patients were referred from the Department of Cardiology or outpatient clinic, Uppsala University Hospital, for a routine transthoracic echocardiography for different indications and had acceptable acoustic windows. The rate of recruitment was determined by whether two of the three echo examiners (TB, EMH, FAF) could acquire the data at the scheduled time in a patient fulfilling the above criteria. No other selection criteria were applied. The study was approved by the Regional Ethical Review Board at Uppsala University (Reference number: 2013/487).

**Fig. 1:**
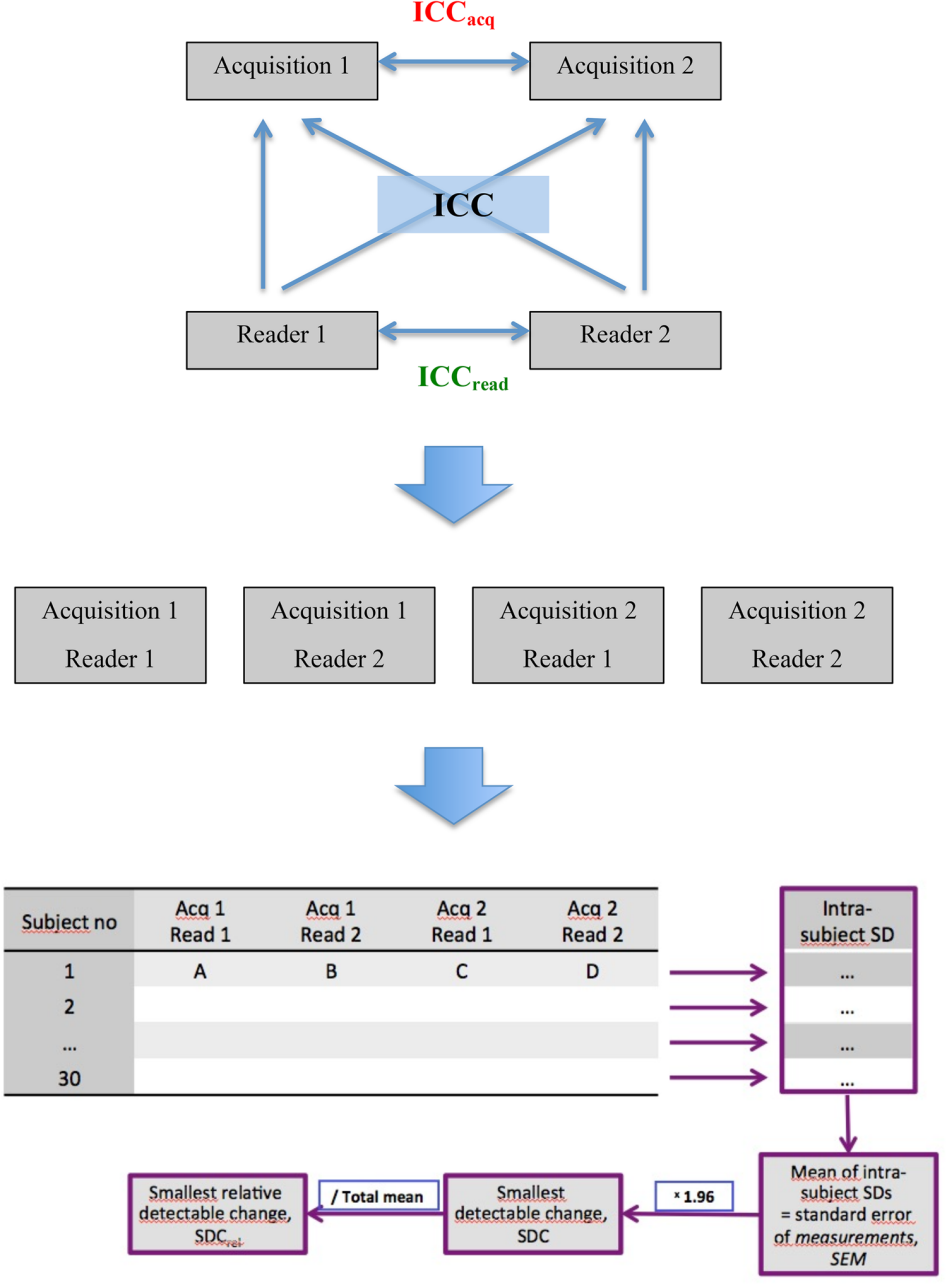
Study design. Standard echo sequences were acquired independently by two different, echocardiographers. Both image sets (acquisition 1 and 2) were then analyzed by two independent readers creating 4 data sets per patient (acquisition 1/reading 1, acquisition 1/reading 2, acquisition 2/reading 1 and acquisition 2/reading 2). Intra-class Correlation Coefficient (ICC) for single measures was calculated globally for the 4 image sets and then separately for two different acquisitions analyzed by the same reader (inter-acquisition reliability, ICC_acq_) and two different readers analyzing the same acquisition (inter-reader reliability, ICC_read_). For estimation of intra-subject variability, the standard deviation of the four measurements of each parameter in each patient was calculated. The mean of these intra-subject standard deviations resulted in the standard error of measurements (SEM) for the whole studied group. The smallest detectable change (SDC) is then calculated as 1.96 × SEM, representing the minimal difference between the measurements that must be overcome to ascertain a true change or difference with a less than 5% chance of error. The smallest relative detectable change (SDC_rel_) is defined as the ratio of the SDC to the mean value of the measured parameter

Standard two-dimensional apical four-, three- and two-chamber views, as well as a parasternal mid-papillary short axis view, were acquired independently and in a blinded fashion by two different, experienced echocardiographers in immediate sequence during the same examination, according to the study protocol. Both image sets (acquisition 1 and 2) were analyzed off-line by two independent readers blinded to each other´s results, creating 4 data sets per patient (acquisition 1/reading 1, acquisition 1/reading 2, acquisition 2/reading 1 and acquisition 2/reading 2). In total, 60 acquisitions and 120 analyses were performed; see Fig. 1 for study design. LV end-diastolic volume (EDV), end-systolic volume (ESV) and ejection fraction (EF) were assessed using the biplane Simpson’s method [9]. Mitral annulus plane systolic excursion (MAPSE) was measured by M-mode echocardiography in the apical four chamber view. With the M-mode cursor aligned parallel to the LV walls, the systolic excursion of the mitral annulus was measured from the lowest point at end-diastole to the highest point during systole in the septum and lateral wall, and a mean value was calculated [10].

**Fig. 2:**
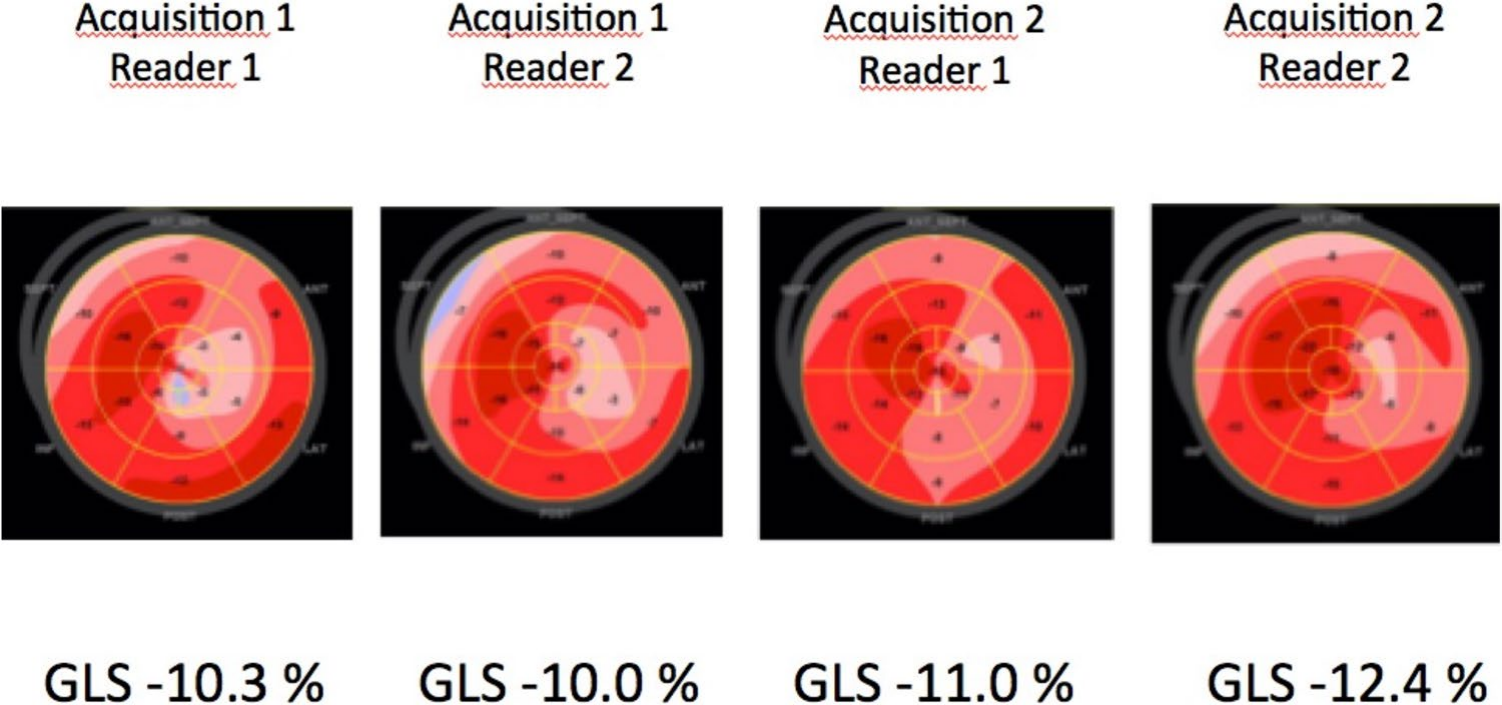
An example of GLS measurements performed in one of the study patients (2 acquisitions taken during the same examination, analyzed off-line by 2 different readers)

All views were obtained at a frame rate of at least 40 frames/s, recording three sequential sinus beats. After choice of the best beat, the endocardial borders were manually traced in the end-diastolic frame for automatic calculation of peak systolic global longitudinal strain (GLS) and circumferential strain (CS), and the resulting region of interest was visually checked for accurate tracking. For GLS calculation, the apical four-, three- and two-chamber views were used, while for CS the mid-ventricular short axis view was traced. We accepted automatic definition of aortic valve closure (AVC) by the software, on the basis of ECG-trigging. Echocardiographic images were acquired with a GE Vivid E9 Ultrasound system. According to recommendations from the American Society of Echocardiography and the European Association of Cardiovascular Imaging we excluded images with suboptimal tracking of the endocardium in more than two segments in one single view or if frame rate was below 40 Hz. Figure 2 presents an example of GLS measurements performed in one of the study patients.

### Statistical analysis

For sample size calculation, we assumed that intra-class correlation coefficients (ICC) would be 0.9 and required that the margin of error of a 95% confidence interval (CI) for ICC should be 0.07, i.e., the CI should be the estimated ICC ± 0.07. These conditions indicated a sample size of 30 patients.

Categorical variables were presented as the number of patients and percentages. Continuous variables were presented as mean ± standard deviation (SD). From 2 acquisitions (acquisition 1 and 2) and 2 readings (reading 1 and 2) a total of 4 data values per patient were obtained.

Reliability of measurements was measured in two ways (see Fig. 1):

1. *Smallest detectable change (SDC)* The square root of the intra-subject variability, which is the standard deviation of the four measurements within patients (standard error of measurements, SEM), was calculated. The SDC was then calculated as 1.96 × SEM, representing the minimal difference between the measurements that must be overcome to ascertain a true change or difference with a less than 5% chance of error. To enable comparison between parameters, the relative smallest detectable change (SDC_rel_) was defined as the ratio of the SDC to the mean value of the measured parameter.
2. *Intra-class correlation coefficient (ICC)* for single measures. The ICC is defined as the ratio of the variance between subjects to the total variability which is the sum of between-subject and intra-subject variability. These variance components were estimated in a one-way analysis of variance model with patient as factor. We used Cicchetti’s guidelines for interpretation of ICC values [11].

In order to separate and quantify the impact of acquisition and reading on the reproducibility of measurements, the ICC was calculated separately for two different acquisitions analyzed by the same reader (inter-acquisition reliability, ICC_acq_) and two different readers analyzing the same acquisition (inter-reader reliability, ICC_read_); see Fig. 1. The difference between ICC_acq_ and ICC_read_ for all the analyzed parameters was calculated and presented with 95% confidence interval and *p* value, assessed with jackknife technique.

Furthermore, the mean intra-subject difference between acquisition 1 and 2 after averaging of reading 1 and 2, and the mean difference between reading 1 and 2 after averaging of acquisition 1 and 2, were calculated for all studied parameters, to visualize the effect of acquisition and reading, respectively. Again, the relative mean intra-subject differences were calculated separately for acquisition and reading, by dividing the absolute mean intra-subject difference by the mean value of the respective parameter within the patient, and expressed as percent of the mean within the subject.

For all analyses, two-sided *p* values < 0.05 were defined as statistically significant. Statistical analyses were performed using SAS Software 9.4. (SAS Institute, Cary, NC, USA) and IBM SPSS version 24 (SPSS, IBM Corporation, Armonk, NY, USA).

## Results

Mean age of the study population was 60.6 ± 18.9 years and 63.3% (*n* = 19) of the patients were males. For baseline characteristics see Table 1. Mean EF was 46.4 ± 16.4%, ranged between 17 and 71% and was impaired (below 52% in males and below 54% in females) in 60% (*n* = 18) of the patients. For mean values of the studied parameters in total and in separate data sets see Table 2.

**Table 1:** Baseline characteristics of the study population (*n* = 30)

Variable	Value
Age (years)	60.6 ± 18.9
Women	11 (36.7%)
Body surface area (m^2^)	1.92 ± 0.19
Systolic blood pressure (mmHg)	125 ± 18
Diastolic blood pressure (mmHg)	74 ± 11
Heart rate (bmp)	69 ± 8

Values are *n* (%) or mean ± SD

**Table 2:** Echocardiographic data obtained by reading 1 and 2 of acquisition 1 and 2, mean of all patients’ mean values and range of the measurements (*n* = 30)

Variable	Acquisition 1		Acquisition 2		Mean (SD)^a^	Range
	Reader 1	Reader 2	Reader 1	Reader 2		
Global longitudinal strain (%)	− 11.6 ± 5.2	− 11.5 ± 5.1	− 11.7 ± 4.8	− 11.7 ± 4.9	− 11.6 ± 5.0	− 22.6; − 1.2
Circumferential strain (%)	− 10.8 ± 5.7	− 9.9 ± 4.9	− 10.1 ± 5.1	− 9.3 ± 4.9	− 10.1 ± 5.0	− 23.1; 0
Ejection fraction (%)	47.5 ± 16.7	46.6 ± 17.0	46.3 ± 16.3	45.3 ± 16.8	46.4 ± 16.4	14; 73
End-diastolic volume (ml)	143.0 ± 62.3	138.5 ± 61.7	143.1 ± 62.3	137.1 ± 64.2	140.4 ± 61.9	58; 262
End-systolic volume (ml)	82.1 ± 55.8	81.5 ± 57.0	83.9 ± 55.8	82.8 ± 58.6	82.6 ± 56.4	17; 212
Mitral annular plane systolic excursion (mm)	10.4 ± 2.9	10.2 ± 2.9	10.2 ± 3.0	10.2 ± 2.9	10.3 ± 2.9	5.5; 17.0

Values are mean ± SD

^a^Calculated as mean ± SD of all patients’ mean values

The relative smallest detectable change among the conventional echo parameters was lowest for MAPSE (10.7%). Among strain parameters, the relative smallest detectable change was much smaller for GLS than for CS (14.7 and 35.6%, respectively), but similar to the SDC_rel_ observed for volume-derived EF (14.2%). The intra-class correlation coefficient was excellent for all measures of the left ventricular function. For ICCs, their 95% CI, intra-subject SDs and smallest detectable changes, see Table 3.

**Table 3:** Global reproducibility of LV function measurements (*n* = 30)

Variable	Mean intra-subject SD	SDC	SDC_rel_	ICC	95% CI
Global longitudinal strain	0.85%	1.7%	14.7%	0.985	0.978–0.991
Circumferential strain	1.82%	3.6%	35.6%	0.931	0.902–0.959
Ejection fraction	3.35%	6.6%	14.2%	0.979	0.971–0.988
End-diastolic volume	11.1 ml	21.8 ml	15.5%	0.984	0.977–0.990
End-systolic volume	8.1 ml	15.9 ml	19.2%	0.990	0.985–0.994
Mitral annular plane systolic excursion	0.56 mm	1.1 mm	10.7%	0.980	0.971–0.988

*Mean intra-subject SD* mean of the standard deviations of intra-subject measurements, *SDC* smallest detectable change (= 1.96 × mean intra-subject SD), *SDC*_*rel*_ the relative smallest detectable change (= SDC/mean of patients’ mean absolute values of the measured parameter), *ICC* intra-class correlation coefficient;

Intra-patient inter-acquisition reliability (ICC_acq_) was significantly better than inter-reader reliability (ICC_read_) only for EF, while no significant difference between those components of test–retest reliability was observed for any other LV function parameter, see Table 4.

**Table 4:** Inter-acquisition (ICC_acq_) and inter-reader (ICC_read_) reproducibility of LV function measurements (*n* = 30)

Variable	ICC_read_	95% CI	ICC_acq_	95% CI	ICC_read_–ICC_acq_	95% CI diff	*p* value diff
Global longitudinal strain	0.974	0.960–0.988	0.968	0.950–0.985	0.006	− 0.007 to 0.020	0.376
Circumferential strain	0.908	0.839–0.978	0.865	0.771–0.958	0.044	− 0.053 to 0.141	0.381
Ejection fraction	0.950	0.925–0.974	0.984	0.975–0.993	− 0.034	− 0.055 to 0.013	0.003
End-diastolic volume	0.966	0.941–0.991	0.979	0.970–0.989	− 0.013	− 0.036 to 0.010	0.263
End-systolic volume	0.976	0.961–0.990	0.989	0.980–0.997	− 0.013	− 0.027 to 0.001	0.075
Mitral annular plane systolic excursion	0.968	0.944–0.992	0.958	0.927–0.988	0.010	− 0.004 to 0.025	0.180

**Fig. 3:**
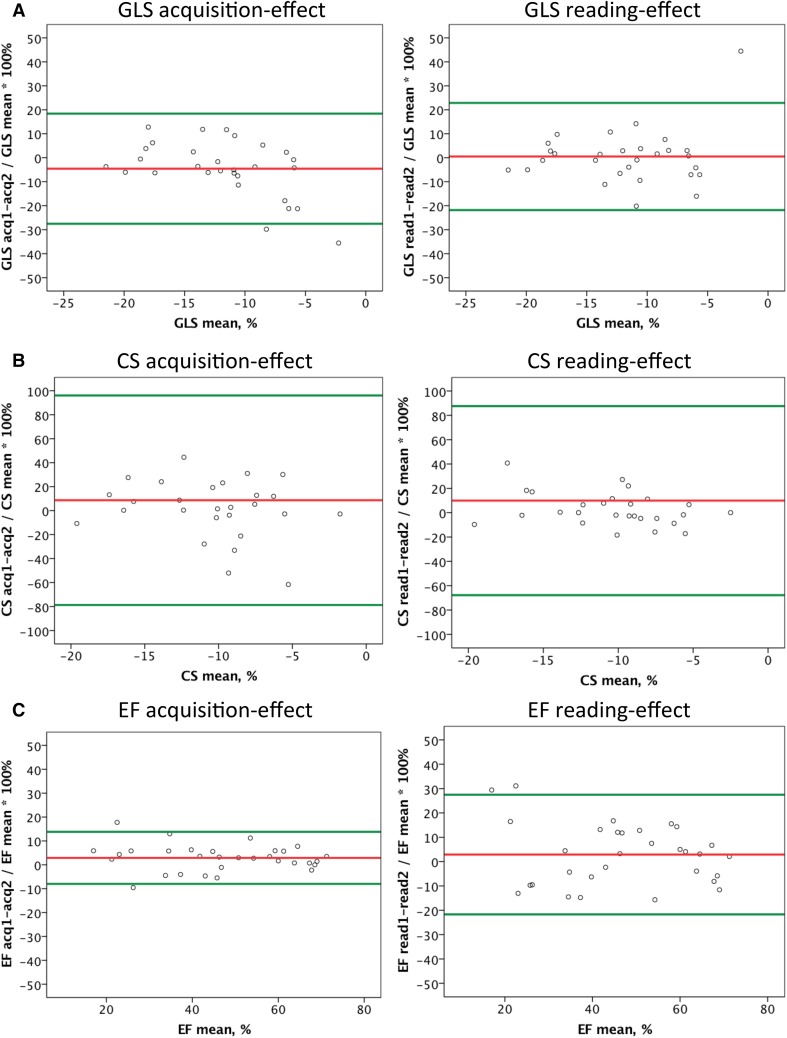

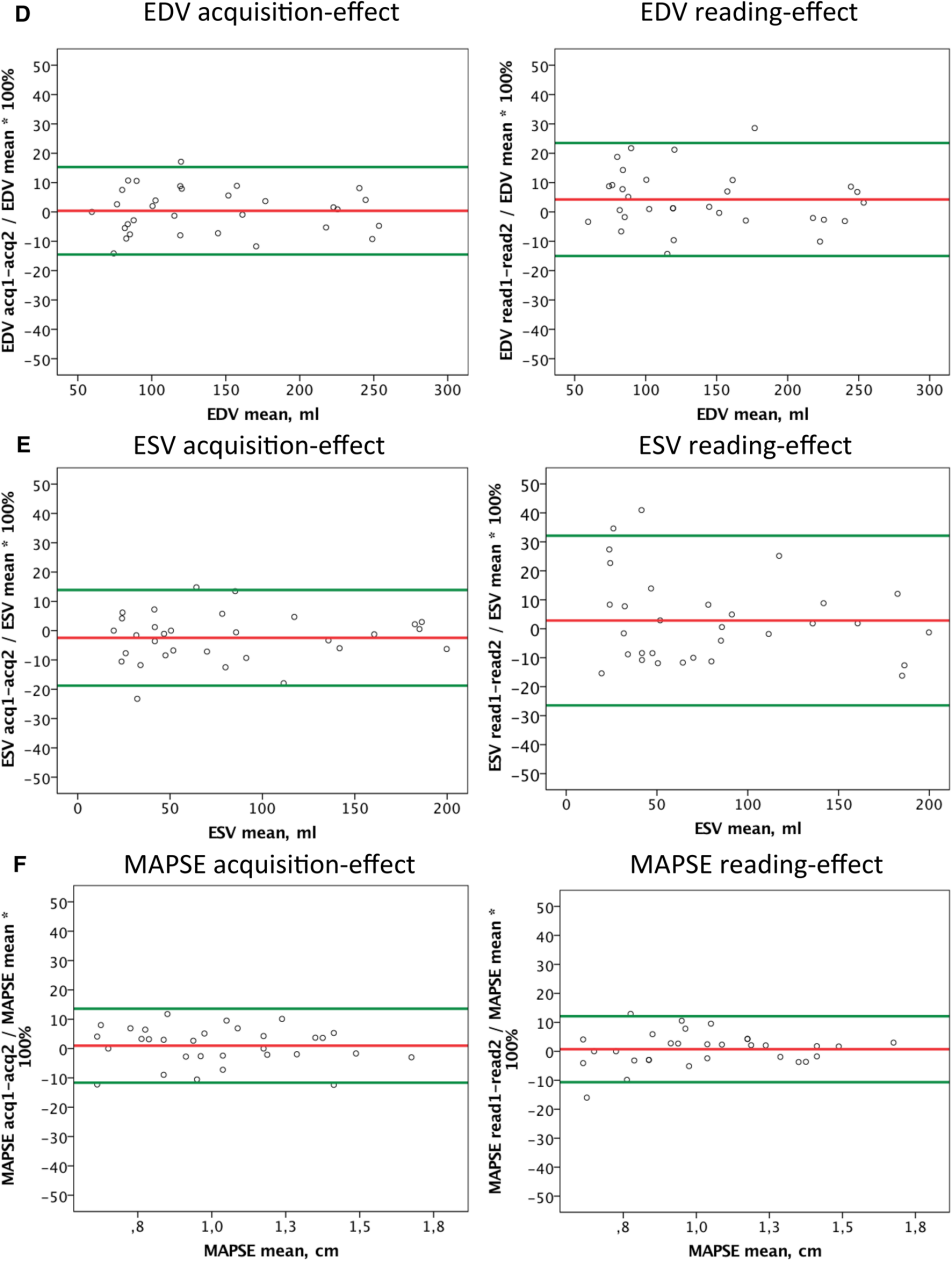
Bland–Altman plots showing the mean intra-subject relative difference (expressed as percent of the mean within the subject) between acquisition 1 and 2 after averaging of reading 1 and 2 for each acquisition (acquisition-effect) and the mean difference between reading 1 and 2 after averaging of acquisition 1 and 2 for each reading (reading-effect) for GLS (**a**), CS (**b**), EF (**c**), EDV (**d**), ESV (**e**) and MAPSE (**f**) versus the mean value of the respective parameter within the patient. Colored lines indicate bias and limits of agreement (1.96 × SD)

Mean intra-subject relative difference (expressed as percent of the mean within the subject) between acquisition 1 and 2 after averaging of reading 1 and 2 for each acquisition (acquisition-effect) and the mean difference between reading 1 and 2 after averaging of acquisition 1 and 2 for each reading (reading-effect) for strain parameters, volume-derived LV function measurements and MAPSE in relation to the mean value of respective parameter within the patient are presented in Fig. 3.

**Fig. 4:**
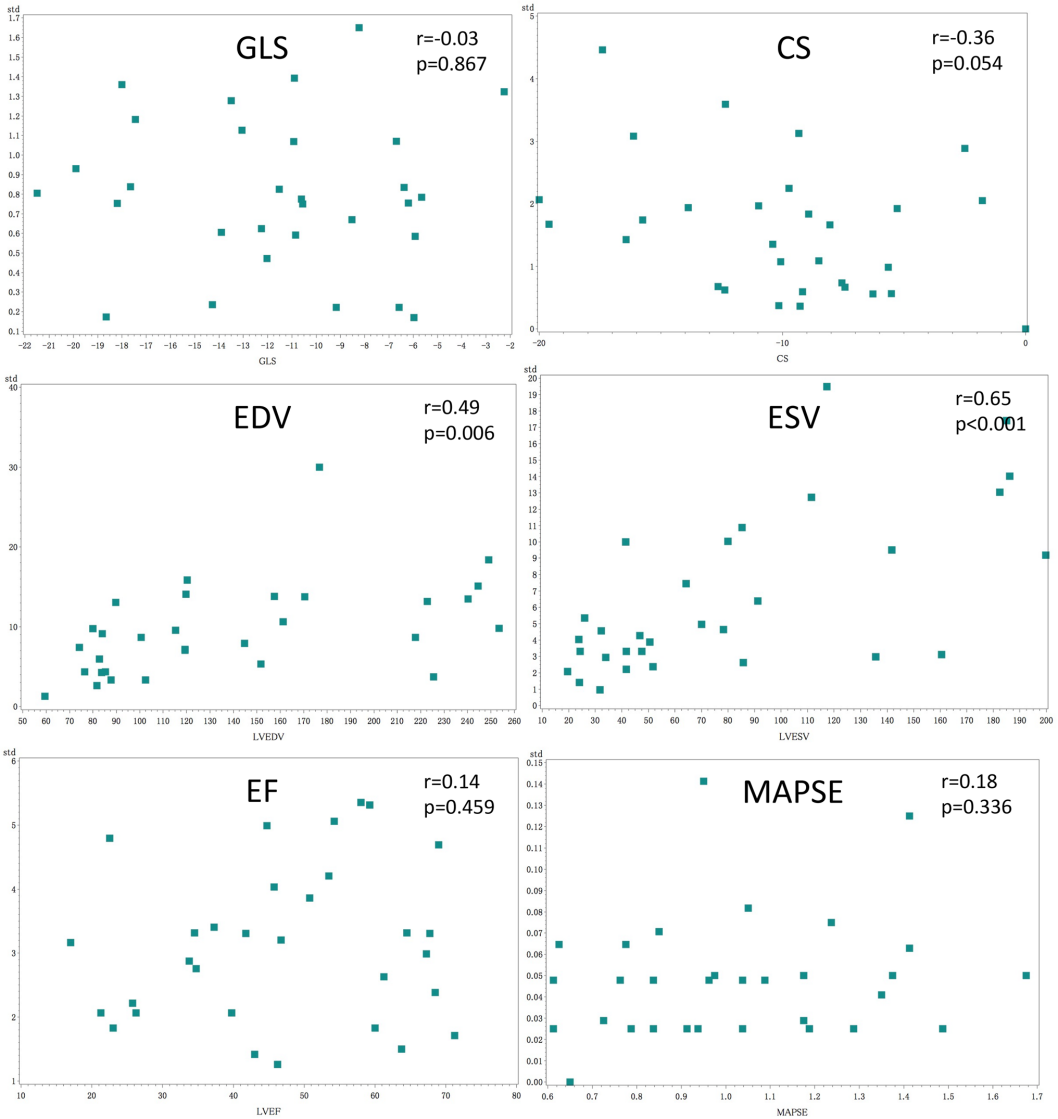
Correlation between mean intra-subject standard deviations of LV function parameters and their absolute values, indicating that the calculated smallest detectable changes are independent (homoscedastic) of LV function impairment level when assessed by GLS, CS, EF and MAPSE. Intra-subject standard deviations significantly correlated with absolute values of LV volumes

With the exception of LV volumes, mean intra-subject standard deviations of EF, MAPSE and GLS did not correlate with their absolute values, indicating that the calculated smallest detectable changes could be shown to be independent (homoscedastic) of LV function impairment level; see Fig. 4.

## Discussion

In the present study, systematically evaluating components of test–retest reliability in a wide range of left ventricular functions and in a true test–retest clinical setting, we found a reasonable reproducibility for both GLS and conventional measures of left ventricular function. The smallest detectable change of GLS, EF and MAPSE ranged between 11–15% and was not dependent on the grade of LV function impairment. Importantly, GLS measurement did not show higher test–retest reliability than EF. Circumferential strain showed much poorer reproducibility. Repeated recordings had an equal impact on reliability of measurements as repeated readings for all the parameters except for ejection fraction, for which the variability was more dependent on the reader than on the acquisition.

Reproducibility of measurements is pivotal for the clinical application of cardiac imaging, but has received much less attention than accuracy. Measurement data from cardiac imaging, including echocardiography, no matter how accurate, are only meaningful when we can be confident that they are reproducible in other examiners’ hands. However, such measurements inherently and inevitably have considerable measurement variability, which stems from differences in acquisition, in data processing, and in data interpretation, besides the possibility of biological variability of the “true” data, e.g., due to changes in heart rate, blood pressure, or cardiac function itself.

An important component of measurement variability is test–retest reliability (also called reproducibility, repeatability, or robustness), which describes variability of separately acquired and interpreted echocardiographic measurements of the same patient, independent of “true” underlying biological variability. This is the typical clinical scenario for example for follow-up examinations or re-examinations of referred patients. The amount of test–retest reliability must be known in order to decide, with confidence, whether a recorded difference in functional parameters represents a “true” change in cardiac function or just reflects measurement variability.

Echocardiographic measurements of left ventricular function, in particular left ventricular ejection fraction, have been known to involve substantial measurement variability. However, the vast majority of reported “inter-observer” and “intra-observer” variabilities reflect only the variability between different observers reading the same dataset, or of one observer reading the same dataset repeatedly. True test–retest reliability is clinically more meaningful, because it reflects more closely the reality of healthcare, but such data are comparatively scarce.

### Reliability of conventional echo parameters of LV function

Variability of conventional echo parameters in a test–retest setting was studied by Otterstad et al. [12], who identified repeated acquisitions as the major component of variability of left ventricular volumes and mass, followed by variability between different readers (inter-observer variability), or between serial readings of the same examination (intra-observer variability). Coefficients of variation due to repeated registrations were 11.6, 7.5 and 12.2% for EDV, ESV and EF, respectively. The smallest detectable change taking into account variation from different readers and repeated readings by the same reader was 16.3, 20.0 and 18.1%, respectively, corresponding with our observations.

Similar test–retest reliability data from early 2D echocardiography were reported by Gordon et al. [13], who showed that the smallest intra-subject detectable change was 15% for EDV, 25% for ESV and 10% for EF. The follow-up acquisitions were however not performed on the same day, so that biologic variability was a possible confounding factor.

Thavendiranathan et al. studied 56 females undergoing chemotherapy at 2 different time points assuming unchanged LV function between measurements evaluated by GLS. The authors reported the smallest detectable change (calculated analogously to our study as 2 × standard error of measurements) of absolute 13% for EF, 59 ml for EDV and 29 ml for ESV [14], which is almost two times higher than in our study. This can probably be explained by expected biological variability between the acquisitions.

### Speckle-tracking-derived strain analysis

Less reproducibility data than for volume-based measurements are available for longitudinal strain measurements including GLS. Although intra-observer and inter-observer variability of measurements performed on the same dataset has been assessed in many studies, few data on a true test–retest reliability exist, and these data are largely from patients with normal or near normal left ventricular function [15–18].

Farsalinos et al. in the largest to date study designed for head-to-head comparison of GLS measurements among seven different vendors, examined a group of 62 volunteers with normal/near normal LV systolic function (average EF 60%) [18]. During the same day the participants were scanned twice by the same echocardiographer and once by another one and the exams were subsequently read, contrary to our study, by the same single physician. Inter-observer relative mean error (mean difference of GLS between acquisitions in percent of mean of absolute measurements), was in average 6.9% for GLS and 10.1% for EF. The parameter “relative mean error” used in Farsalinos’ study analytically corresponds to half the value of the relative SDC (SDC_rel_) reported in our study, and the results are similar in case of GLS (SDC_rel_ 14.7%, equivalent to “relative mean error” of 7.4% for our study *vs*. 6.9% in Farsalinos’ study), while the variability of EF is lower (SDC_rel_ 14.2%) in our study, equivalent to “relative mean error” of 7.1% for our study vs. 10.1% in Farsalinos’ study.

Kleijn et al. studied test–retest reliability of both volumetric parameters of LV function and 3 different global strain components, using three-dimensional speckle-tracking echocardiography. In a group of 50 patients with normal or mildly impaired EF, ICC for volume-based parameters was good (0.85 for EF), but only moderate for global strain parameters, however poorer for GLS than for GCS (0.66 and 0.85, respectively) [2]. Calculated absolute SDC for EF was 11% and 5.7% for GLS, almost twice poorer for EF and more than threefold poorer for GLS in our 2D study. The differences can be explained by poorer both spatial and temporal resolution of 3D echo, which makes the use of speckle-tracking-derived deformation parameters limited clinically.

Barbier et al. reported test–retest reliability of strain and volume-based LV function parameters, when the same operator in random order read two image sets recorded during the same examination (not a real test–retest situation) in 40 patients with normal/near normal EF (average EF 52%). The variability of global longitudinal strain was slightly lower for GLS than for EF (coefficient of variation of 5.4% and 6.8%, respectively), which is in the range of our results (calculated from our results the coefficient of variation would be 7.3% and 7.2%, respectively). Interestingly, the measurement variability was higher in the lower range of EF, which is in contrast to our study showing no association between grade of measurement variability and LV impairment [1].

Thorstensen et al., in a group of 10 healthy individuals, evaluated impact of repeated recordings (within 30 min) (inter-observer reproducibility) and analyses (inter-analyzer reproducibility) on variability of LV function parameters [15]. Reported coefficients of repeatability, calculated as 2 x mean intra-subject SD, corresponding with the SDC in current study, were in similar range for EF (7% vs. 6.6% in current study) and GLS (2 strain% vs. 1.7 strain%), and slightly higher for MAPSE (1.6 mm vs. 1.1 mm in current study). They reported an equal impact of repeated acquisitions and readings on the measurement variability of EF and GLS, while for MAPSE repeated acquisitions showed slightly higher impact on measurement variability comparing with repeated readings (mean error of 4% vs. 3%, respectively). Contrary, in the current study only the variability of EF measurements was more dependent on the reader than on acquisition, while both these components to a similar extent influenced the variability of all other measurements of left ventricular function.

### Study limitations

Firstly, variability of left ventricular function parameters is strongly influenced by the image quality. In the current study, we included patients with satisfactory quality of the images and with sinus rhythm, therefore the study population is selected. Secondly, the current study was performed using one manufacturer and software. Although only minor, significant inter-manufacturer differences have been described [18, 19], which in practice further compound the problem of test–retest reliability if echo machines or software from different manufacturers are used in different examinations. Thirdly, given the recently disputed accuracy and variability of segmental longitudinal strain [20, 21], as well as a limited size of the study group and its purpose to compare reliability of global systolic function parameters, we address only GLS measurements.

## Conclusion

In a test–retest setting, both with normal and impaired left ventricular function, the smallest relative detectable change of EF, GLS and MAPSE was similar (11–15%), but was much higher for CS (35%). Surprisingly, reliability of GLS was not superior to that of EF. Acquisition and reader to a similar extent influenced the variability of measurements of all left ventricular function measures except for ejection fraction, where variability was more dependent on the reader than on the acquisition.
